# Metformin promotes anti-tumor immunity in *STK11* mutant NSCLC through AXIN1-dependent upregulation of multiple nucleotide metabolites

**DOI:** 10.32604/or.2024.052664

**Published:** 2024-09-18

**Authors:** ZHIGUO WANG, KUNLIN LI, CONGHUA LU, MINGXIA FENG, CAIYU LIN, GUOFANG YIN, DAN LUO, WENYI LIU, KAIYU JIN, YUANYAO DOU, DI WU, JIE ZHENG, KEJUN ZHANG, LI LI, XIANMING FAN

**Affiliations:** 1 Department of Respiratory and Critical Care Medicine, Affiliated Hospital of Southwest Medical University, Luzhou, 646000, China; 2 Department of Respiratory Disease, Daping Hospital, Army Medical University (Third Military Medical University), Chongqing, 400042, China; 3 Department of Trauma Medical Center, Daping Hospital, State Key Laboratory of Trauma, Burns and Combined Injury, Army Medical University, Chongqing, 400042, China; 4 Department of Respiratory Disease, People’s Hospital of Xuyong County, Luzhou, 646000, China; 5 Department of Outpatients, Daping Hospital, Army Medical University (Third Military Medical University), Chongqing, 400042, China

**Keywords:** Metformin, Serine/threonine kinase 11 *(STK11)*, Lung cancer, Axis inhibition protein 1 (AXIN1), Nucleotide metabolites

## Abstract

**Background:**

Metformin has pleiotropic effects beyond glucose reduction, including tumor inhibition and immune regulation. It enhanced the anti-tumor effects of programmed cell death protein 1 (PD-1) inhibitors in serine/threonine kinase 11 (*STK11)* mutant non-small cell lung cancer (NSCLC) through an axis inhibition protein 1 (AXIN1)-dependent manner. However, the alterations of tumor metabolism and metabolites upon metformin administration remain unclear.

**Methods:**

We performed untargeted metabolomics using liquid chromatography (LC)-mass spectrometry (MS)/MS system and conducted cell experiments to verify the results of bioinformatics analysis.

**Results:**

According to the Kyoto Encyclopedia of Genes and Genomes (KEGG) pathway database, most metabolites were annotated into metabolism, including nucleotide metabolism. Next, the differentially expressed metabolites in H460 (refers to H460 cells), H460_met (refers to metformin-treated H460 cells), and H460_KO_met (refers to metformin-treated *Axin1*^*-/-*^ H460 cells) were distributed into six clusters based on expression patterns. The clusters with a reversed expression pattern upon metformin treatment were selected for further analysis. We screened out metabolic pathways through KEGG pathway enrichment analysis and found that multiple nucleotide metabolites enriched in this pathway were upregulated. Furthermore, these metabolites enhanced the cytotoxicity of activated T cells on H460 cells *in vitro* and can activate the stimulator of the interferon genes (STING) pathway independently of AXIN1.

**Conclusion:**

Relying on AXIN1, metformin upregulated multiple nucleotide metabolites which promoted STING signaling and the killing of activated T cells in *STK11* mutant NSCLC, indicating a potential immunotherapeutic strategy for *STK11* mutant NSCLC.

## Introduction

Lung cancer, the most common cancer, was also the leading cause of cancer deaths worldwide [1]. In the last decade, immune checkpoint inhibitors (ICIs) targeting the programmed cell death protein 1 (PD-1)/programmed cell death-ligand 1 (PD-L1) axis had produced remarkably durable responses in non-small cell lung cancer (NSCLC) which accounts for about 85% of all lung cancers, leading to prolonged survival in select patients [2,3]. Unfortunately, a high incidence of primary and acquired resistance to ICIs, has become an urgent problem limiting its clinical applications. Serine/threonine kinase 11 (*STK11*) is a tumor suppressor gene that encodes the Liver Kinase B1 (LKB1) protein. Mutation of *STK11* accounts for 20% of lung adenocarcinoma (LUAD), and confers peculiar characteristics to NSCLC, including disordered cellular metabolism, inactivation of stimulator of interferon genes (STING), and resistance to immunotherapy [4–7]. Strikingly, *STK11/LKB1* mutations have been implicated as potential drivers of primary resistance to ICIs [6,8]. Interestingly, changes in tumor cell metabolism driven by oncogenes or tumor suppressor genes can impact the tumor microenvironment (TME) and immune responses against cancer. Based on the importance of metabolism immunity, it is of interest to the field to explore how the TME shapes immunity against *STK11* mutant NSCLC to develop new strategies to improve the efficacy of ICI treatment.

Mounting evidence has suggested that metformin can exert an anti-tumor effect in various cancers, such as lung cancer, prostatic cancer, and colon cancer [9,10]. Studies have found that metformin can potentially target *STK11* mutant lung cancer alone or in combination with conventional chemo/radiotherapy [11–13]. Furthermore, it has been reported that metformin can potentiate anti-tumor immunity by inducing the reduction of tumor hypoxia [14], and via activation of the nuclear factor E2-related factor 2 (Nrf2)/mammalian target of rapamycin complex 1(mTORC1)/p62 axis in tumor-infiltrating CD8 T cells [15]. Additionally, we previously demonstrated that combinatorial treatment with metformin and pembrolizumab enhanced anti-tumor immunity in *STK11* mutant lung cancer via axis inhibition protein 1 (AXIN1)-dependent inhibition of STING ubiquitination [16]. However, given metformin’s role as a regulator of metabolism, understanding how metformin affects the metabolism of *STK11* mutant NSCLC, and whether this process is involved in promoting anti-tumor immunity remains unknown.

The TME, a complex network of various cytokines, chemokines, immune cells, fibroblasts, interstitial cells, and other metabolites, is essential in regulating tumor immunity. Abnormal cell metabolism and metabolites in the TME, such as glucose and amino acid metabolism, adenosine production, and sphingosinol metabolism, can significantly affect immune function and result in a failure of anti-tumor immunity [17]. Cyclic dinucleotides (CDNs), a class of STING agonists, can activate STING and induce immune responses by reversing the immunosuppressive TME [18]. Of note, it has been reported that the *STK11* mutation was involved in metabolic reprogramming of cancer cells [19,20], and metformin modulates metabolism predominantly through AXIN1-dependent activation of AMP-activated protein kinase (AMPK) [21,22]. Bioinformatic approaches and developments in instrumentation technologies, such as ultra-performance liquid chromatography (UPLC) and mass spectrometry (MS), have improved our understanding of the metabolome [23]. By analyzing small molecular substances in samples, a considerable number of metabolomic studies in NSCLC have been conducted, providing in-depth insight into the molecular mechanisms of disease [24]. Our previous work demonstrated that metformin promoted the anti-tumor effect of PD-1 inhibitor in H460 cells, which have a nonsense *STK11* mutation naturally, but not in *Axin1*^*-/-*^ H460 cells [16]. Thus, we aimed to explore the mechanisms governing enhanced anti-tumor immunity induced by metformin through untargeted metabolomic approaches.

## Materials and Methods

### Cell culture and reagents

The human NSCLC cell line H460, which has a nonsense *STK11* mutation at codon 37, was purchased from the American Type Culture Collection (ATCC). The *Axin1*^-/-^ H460 cells were generated previously by CRISPR/Cas9 [16]. All cells which didn’t have mycoplasma infection were cultured in RPMI-1640 (Hyclone, Logan, UT, USA) supplemented with 10% fetal bovine serum (FBS, Gibco, Carlsbad, CA, USA) and 1% penicillin/streptomycin at 37°C in a humidified 5% CO_2_ incubator. Antibodies against STING, TBK1, IRF3, p-TBK1, p-IRF3, and β-Actin were from Cell Signaling Technology (Boston, MA, USA). Metformin was purchased from Selleck (Houston, TX, USA). Adenosine diphosphate (ADP) and uridine diphosphate (UDP)-N-acetylglucosamine were purchased from Solarbio (Beijing, China), while the ADP assay kit, cytidine diphosphate (CDP) and deoxythymidine monophosphate (dTMP) were from Abcam (Cambridge, UK).

### Generation of activated T cells

We acquired the activated T cells as a previous study reported [25]. Briefly, human peripheral blood T cells (SAILYBIO, Shanghai, China) were cultured in ImmunoCult-XF T cell expansion medium supplemented with interleukin-2 (IL-2) (10 ng/mL; PeproTech, Cranbury, NJ, USA) and ImmunoCult Human CD3/CD28/CD2 T cell activator (25 μl/mL; STEMCELL Technologies, Vancouver, CA, Canada). All experiments were conducted in DMEM/F12 medium with IL-2 (10 ng/mL) and anti-CD3 antibody (100 ng/mL; eBioscience, Thermo Scientific, Waltham, MA, USA).

### Cell viability assay

Cell Counting Kit-8 (CCK8; MedChemExpress, Monmouth Junction, NJ, USA) was used to determine the cell viability according to the manufacturer’s instructions. In brief, cancer cells were incubated in a 96-well plate overnight, then treated as indicated for 48 h. Next, the medium was replenished, followed by measuring the absorbances at 450 nm on a Sunrise R microplate reader (Thermo Fisher Scientific, Waltham, MA, USA).

### Immunofluorescence (IF) staining

Immunofluorescence staining was executed to detect STING expression. Cell proliferation was measured by Ki67 staining. Briefly, cancer cells were seeded in six-well plates, then treated as indicated for 48 h. Next, samples were fixed with 4% paraformaldehyde for 30 min, permeabilized with 0.25% Triton for 20 min, and blocked with 10% goat serum for 1 h. Then incubated overnight with STING antibody or Ki67 (Proteintech, Wuhan, China). After incubation with secondary antibody and 4,6-diamino-2-phenyl indole (DAPI, Beyotime Biotechnology, Shanghai, China), samples were photographed under a fluorescence microscope.

### Enzyme-linked immunosorbent assay (ELISA)

Interferon (IFN)-γ and IFN-β levels were assessed using Human IFN-γ (Solarbio, Beijing, China) and Human IFN-β (FineTest^®^, Wuhan, China) ELISA KIT, respectively. Briefly, conditioned medium from different treatment groups was collected and detected according to the operation manual.

### Western blot

Samples were harvested from six-well plates by scraping after washing with Phosphate Buffer Saline (PBS), then lysed at 4°C in Radio Immunoprecipitation Assay (RIPA) buffer (Sigma, Aldrich, France). After centrifugation for 20 min (min) at 4°C, the Quick Start™ Bradford (QSB) protein assay kit (Bio-Rad, Hercules, CA, USA) was utilized to determine the protein concentrations. Then, samples were separated on a 10% SDS–PAGE and transferred onto PVDF membranes (Millipore, Darmstadt, German). Subsequently, membranes were blocked with 5% non-fat milk at room temperature, and incubated overnight with primary antibodies. At last, the blots were incubated with secondary antibodies after washing with 1X PBST buffer, and developed by enhanced chemiluminescence using an ECL kit (MedChemExpress, Monmouth Junction, NJ, USA).

### Metabolite extraction

Cells were cultured in a six-well plate at a density of 5 × 10^5^ per well for 48 h with or without metformin (1 mmol/L). Then, cell samples were collected and placed in Eppendorf (EP) tubes, resuspended in pre-chilled 0.1% formic acid and 80% methanol and vortexed. Next, samples were thawed on ice and tubes were agitated for 30 s. After sonification and centrifugation, cell supernatants were freeze-dried and dissolved in 10% methanol. Finally, the solution was injected into the LC-MS/MS system for analysis [26].

### UHPLC-MS/MS analysis

UHPLC-MS/MS analyses were conducted using a Vanquish UHPLC system (Thermo Fisher, Bremen, Germany) combined with an Orbitrap Q Exactive^TM^ HF-X mass spectrometer (Thermo Fisher, Bremen, Germany) at Novogene Co., Ltd. (Beijing, China). Samples were injected onto a Hypesil Gold column with a flow rate of 0.2 mL/min. The eluents for the negative or positive polarity mode were 5 mM ammonium acetate (pH 9.0) and methanol, or 0.1% formic acid in water and methanol. The Q Exactive^TM^ HF-X mass spectrometer was operated in negative/positive polarity mode with a capillary temperature of 320°C (608°F), a spray voltage of 3.2 kV, and the flow rate of sheath and auxiliary gas maintained at 10 arbitrary units (arb) and 40 arb, respectively.

### Data processing and metabolite identification

The raw data files, generated by UHPLC-MS/MS, were processed to perform peak alignment and picking, and quantitation for each metabolite using the Compound Discoverer 3.1 (Thermo Fisher, Bremen, Germany). Then, peak intensities were normalized to the total spectral intensity. According to additive and fragment ions, and molecular ion peaks, the normalized data were utilized to predict the molecular formula of detected metabolites. Then, to obtain accurate qualitative and relative quantitative results, the peaks were matched with the mzCloud (https://www.mzcloud.org/). The data has been deposited in the jianguoyun (https://www.jianguoyun.com/p/DRtao8wQ9M_jDBjkv84FIAA) (24 July 2024).

### Data analysis

Statistical analyses were performed using R version 3.4.3, Python 2.7.6, and CentOS release 6.6. Using the area normalization method, normal transformations were attempted when data were not normally distributed. Principal component analysis (PCA) and Partial least squares discriminant analysis (PLS-DA) were performed using metaX (a flexible and comprehensive software). Univariate analysis (*t*-test) was applied to calculate the statistical significance (*p*-value). Differential metabolites were determined by variable influence on projection (VIP) > 1, and *p*-value < 0.05, and fold change (FC) ≥ 2 or FC ≤ 0.5. Volcano plots were used to evaluate metabolites of interest based on log_2_(FC) and –log_10_(*p*-value) values of each metabolite. For clustering heat maps, data were normalized using z-scores of the intensity areas of differential metabolites and plotted using the pheatmap package in R. Pathway enrichment analysis was performed using the KEGG database. Values of *p* < 0.05 were considered significant. Cytoscape 3.9.1 and Metascape, a plugin of Cytoscape, were used for building a metabolite network.

## Results

### Cluster analysis of LC-MS data

**Figure 1:**
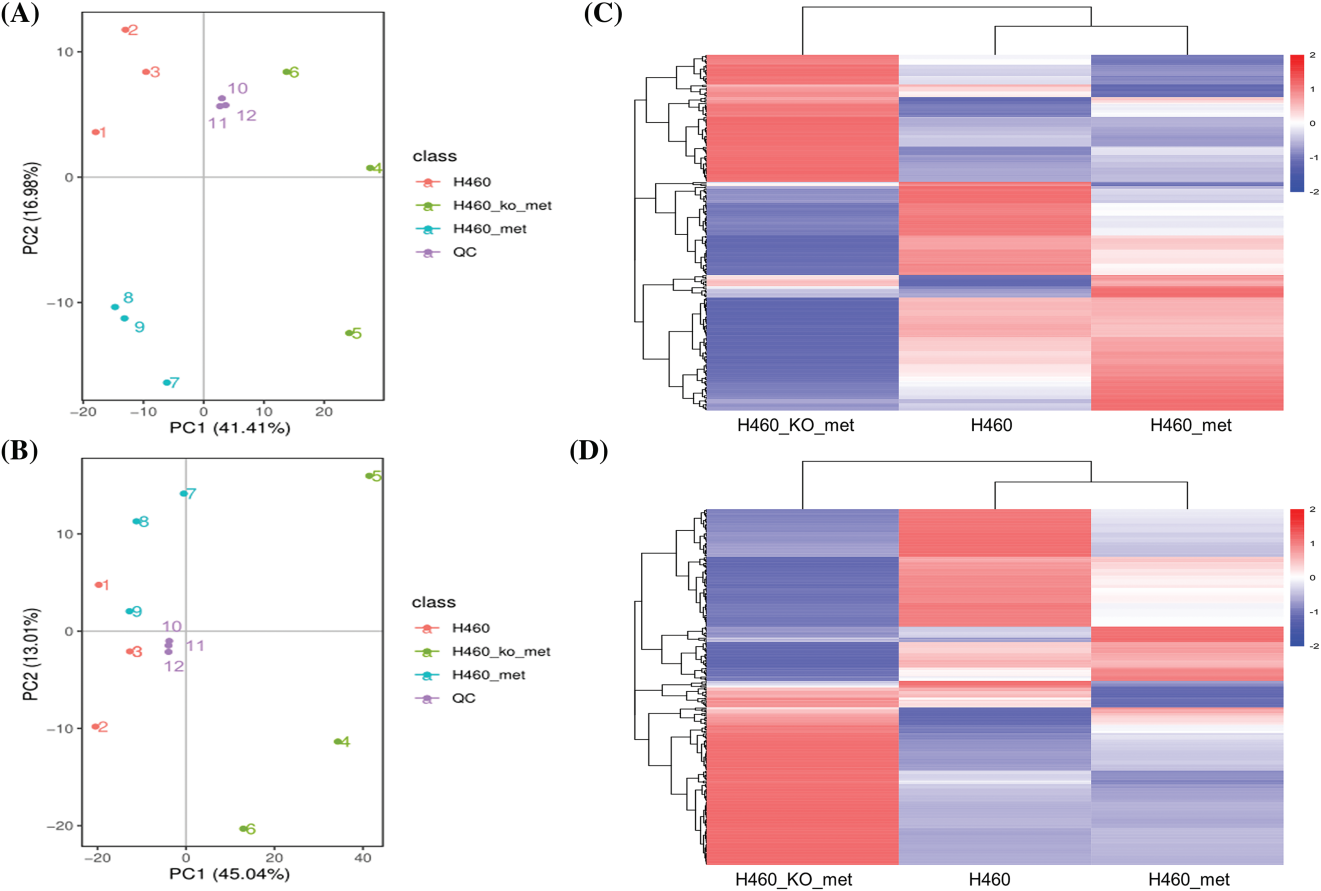
The cluster analysis of differential metabolites. PCA score plot of cell metabolite clustering in H460, H460_met, H460_KO_met, and quality control (QC) groups in negative (A) and positive (B) ion mode, respectively. Cluster analysis of differentially expressed metabolites among the three groups in negative (C) and positive (D) ion mode, respectively. Key: H460: H460 cell samples; H460_met: metformin-treated H460 cell samples, H460_KO_met: metformin-treated *Axin1*^-/-^ H460 cell samples.

To explore the mechanism by which metformin induced anti-tumor immunity in H460 cells but not *Axin1*^-/-^ H460 cells, we first set out to determine differences in tumor metabolism among H460, H460_met, and H460_KO_met cells. The cell samples were analyzed in negative and positive ion modes by LC/MS. Initially, the trajectory analysis of the PCA score plots for H460, H460_met, and H460_KO_met groups showed a clear separation in the negative (Fig. 1A) and positive ion mode (Fig. 1B), respectively. As expected, quality control (QC) samples were predominantly concentrated in a certain zone, demonstrating the stability of the instrument and the reliability of the data. In addition, Pearson correlation analysis between the negative (Fig. S1A) and positive QC samples (Fig. S1B) was applied to verify the quality of the data, demonstrating that the metabolic profiling data obtained were reliable and adequate for further analysis. According to the LC-MS data, a total of 567 and 880 metabolites were identified in negative and positive ion modes respectively, with 252 and 321 differentially expressed metabolites identified among the three groups. Notably, cluster analysis indicated that the metabolites isolated from H460, H460_met, and H460_KO_met groups could be easily distinguished from each other and formed specific clusters. Furthermore, the metabolites in each sample were highly homogeneous within each group regardless of the ion mode (Fig. 1C,D). Cumulatively, These results indicate that our LC/MS approach could identify distinct metabolite characteristics between the three treatment groups, thus allowing us to explore the mechanism of metformin-induced anti-tumor immunity in *STK11* mutant NSCLC that had been demonstrated in our previous study [16].

### Annotation of metabolites based on KEGG and human metabolome database (HMDB)

**Figure 2:**
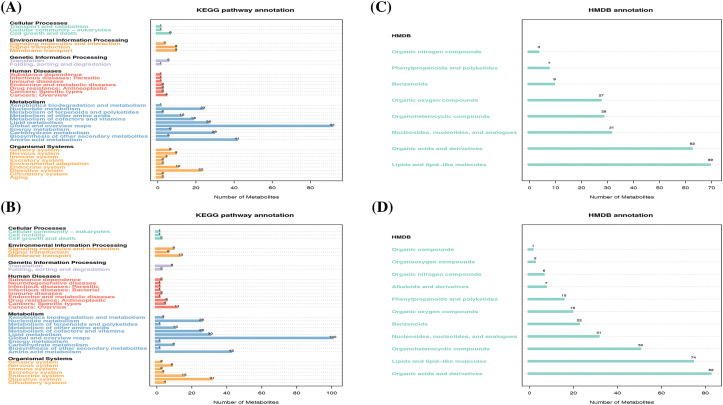
The functional annotation of metabolites according to the KEGG and HMDB databases. KEGG pathway annotation of metabolites in negative (A) and positive (B) ion mode, respectively. HMDB annotation of metabolites in negative (C) and positive (D) ion mode, respectively.

We next conducted functional analysis of the LC/MS data to annotate the identified metabolites and associated metabolic pathways. According to the KEGG pathway database, 169 and 218 metabolites had a corresponding KEGG_ID in the negative and positive ion modes, respectively. As expected, most metabolites were annotated into metabolism which mainly include lipid metabolism, nucleotide metabolism, global and overview maps, carbohydrate metabolism, and amino acid metabolism (Fig. 2A,B). We also performed metabolite annotation using the HMDB. Similarly, 236 metabolites in the negative ion mode and 309 metabolites in the positive ion mode had corresponding HMDB_IDs, and they were dispersed among 8 and 11 super classes based on secondary classification in HMDB. Specifically, the top four classes which have most annotated metabolites both in the negative (Fig. 2C) and positive (Fig. 2D) ion mode are lipids and lipid-like molecules, organic acids and derivatives, organoheterocyclic compounds, as well as nucleosides, nucleotides and analogues. These results indicated that lipid and nucleotide metabolism, the two predominant categories, may mediate anti-tumor immunity during metformin treatment.

### Multiple analytical approaches reveal distinct metabolite differences between H460 and H460_met as well as H460_KO_met and H460_met groups

**Figure 3:**
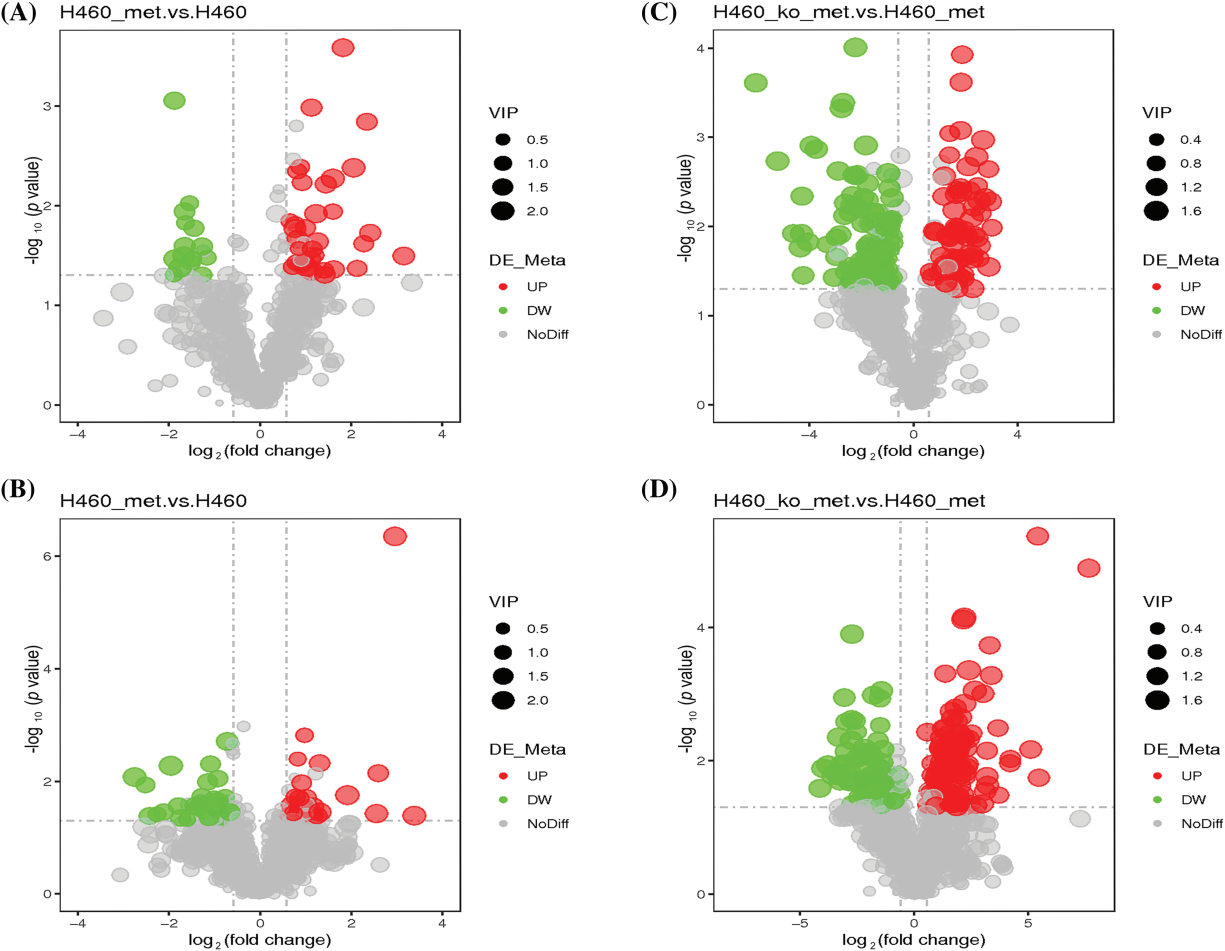
Characterization of the metabolome in H460 and H460_met as well as in H460_KO_met and H460_met cells. Volcano plots showing accumulated [log_2_(FC) on *X* axis] metabolites that were significantly different [–log_10_(*p*-value) on *Y* axis] between the H460 group and H460_met group in negative (A) and positive (B) ion mode, respectively. Volcano plots showing differential metabolites between the H460_KO_met group and H460_met group in negative (C) and positive (D) ion mode, respectively. The green and red points represent respectively down-regulated and up-regulated metabolites.

Next, we analyzed differentially expressed metabolites between H460 and H460_met groups. As shown in Fig. S2A,B, PLS-DA plots demonstrated satisfactory separation between the two groups in both negative and positive ion modes. 49 differentially expressed metabolites were identified in negative ion mode, including 15 down-regulated and 34 up-regulated metabolites. Likewise, in positive ion mode, 54 differential metabolites were identified, including 28 down-regulated and 26 up-regulated metabolites. Differentially expressed metabolites are displayed in volcano plots in the negative (Fig. 3A) and positive (Fig. 3B) ion modes. The specific metabolites and metabolic patterns are illustrated in the metabolite heatmaps (Fig. S3A,B). Similarly, we analyzed differential metabolites between H460_KO_met and H460_met groups. Firstly, PLS-DA plots exhibited a distinct separation between the two groups in both negative (Fig. S2C) and positive (Fig. S2D) ion modes. In addition, 164 and 188 differentially expressed metabolites were screened out in negative and positive ion mode, respectively. More specifically, volcano plots revealed 100 down-regulated (green dots) and 64 up-regulated metabolites (red dots) in negative ion mode (Fig. 3C), and 79 down-regulated and 109 up-regulated metabolites in positive ion mode (Fig. 3D). The metabolite heatmaps depict the metabolic patterns and specific metabolite differences in the negative (Fig. S4A,B) and positive (Fig. S5A,B) ion modes.

### Fuzzy c-means (FCM) clustering analysis of the differential metabolites among the three groups

**Figure 4:**
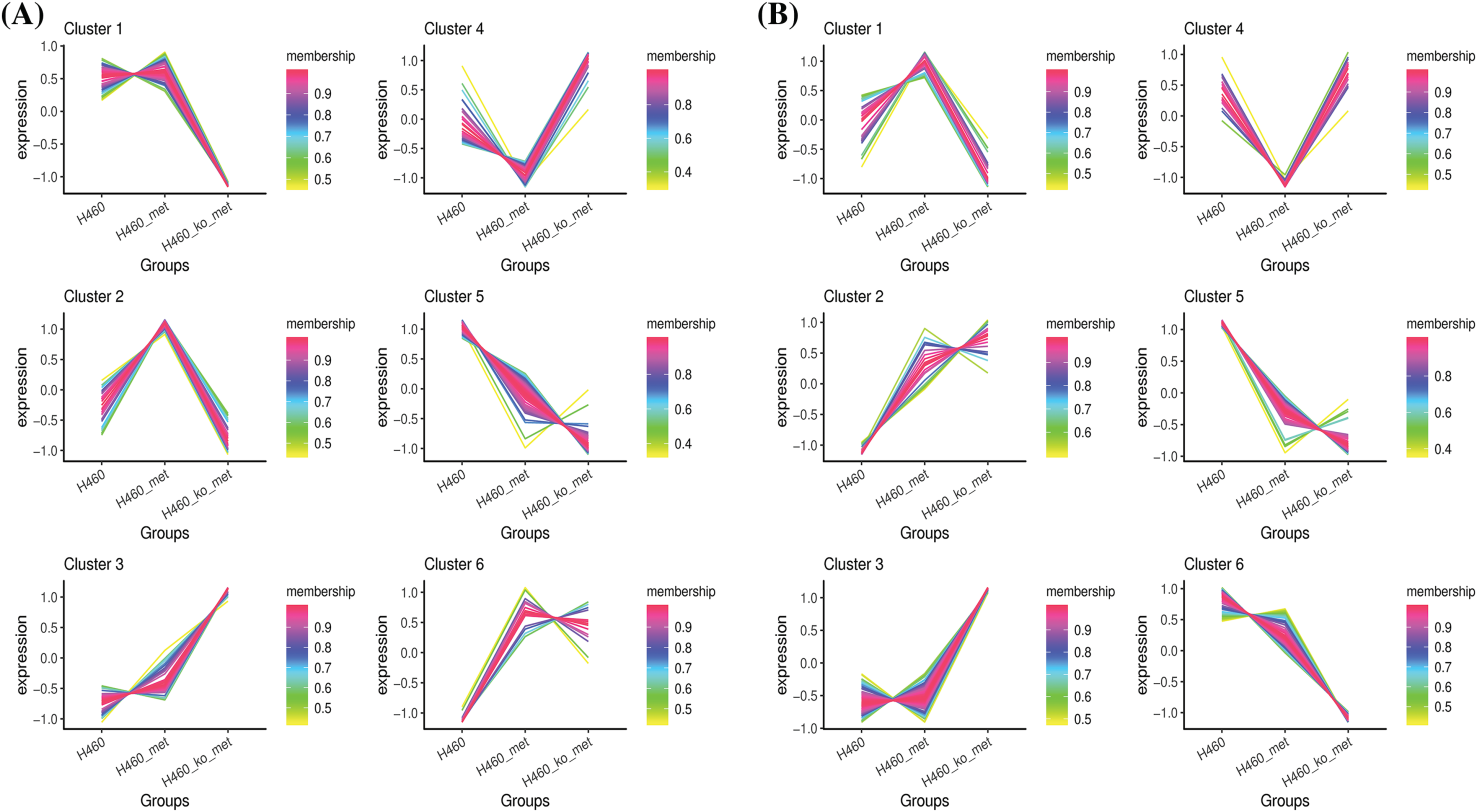
Clustering of the differentially expressed metabolites. The differentially expressed metabolites in negative ion mode (A) and positive ion mode (B) were respectively assigned to six clusters using the FCM clustering algorithm.

Since we considered that metabolites with a reversed expression pattern upon metformin treatment might be involved in promoting anti-tumor immunity, a fuzzy c-means clustering analysis was performed according to the profiles of relative metabolite abundance among the three treatment groups. As shown in Fig. 4A,B, 252 and 321 differential metabolites in negative and positive ion modes were assigned to six clusters. However, we mainly focused our analyses on Cluster 2 (48 metabolites) and Cluster 4 (39 metabolites) in negative ion mode, and Cluster 1 (131 metabolites) and Cluster 4 (67 metabolites) in positive ion mode, in which the levels of metabolites were significantly changed (up or down) after metformin administration and returned to basal levels when *Axin1* was knocked out. Taken together, these results indicated that the crucial metabolites were among the four clusters.

### Analysis of enriched KEGG pathways

**Figure 5:**
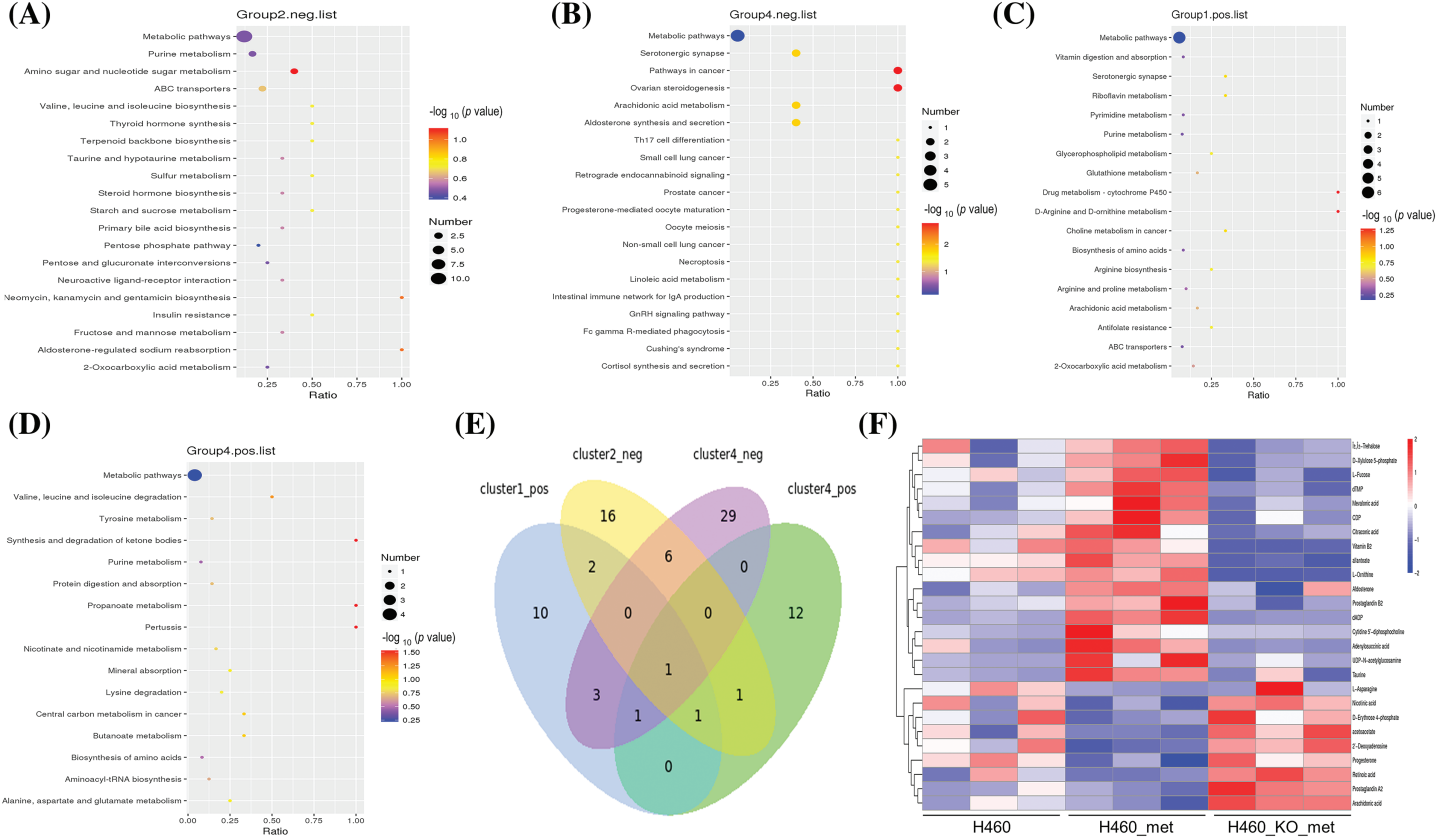
KEGG pathway enrichment analysis. KEGG enrichment scatter plots show the common pathway (metabolic pathways) and other top 19 pathways enriched in Cluster 2 (A) and 4 (B) (negative ion mode). All pathways related to metabolites in Cluster 1 (C) and 4 (D) (positive ion mode) were depicted. (E) Venn diagram of commonly and exclusively differentially expressed pathways, with overall overlapping sites referring to shared pathways in all groups. (F) Heatmap of metabolites enriched in the metabolic pathways among the four selected clusters. The scale represents the expression amount obtained after standardized processing.

To investigate which pathway may lead to enhanced anti-tumor immunity as a result of metformin treatment, we performed KEGG pathway enrichment analysis on candidate metabolites. We identified 27, 40, 18, and 16 pathways in the four selected clusters, respectively. In negative ion mode, the KEGG pathway plot of Cluster 2 and 4 (Fig. 5A,B) revealed more differential metabolites enriched in metabolic pathways, although its *p* value was not the most significant. In Clusters 1 and 4 in positive ion mode, the number of differentially expressed metabolites enriched in the metabolic pathways was also the largest amongst all enriched pathways (Fig. 5C,D). Notably, only one pathway, metabolic pathways, was commonly identified in all clusters. A Venn diagram illustrated the coverage and overlap of pathways (Fig. 5E). Then, we conducted heatmap clustering analysis of all the 26 metabolites enriched in metabolic pathways based on the four selected clusters (Fig. 5F). Thus, these metabolites involved in metabolic pathways may mediate the anti-tumor immunity process of metformin.

### The identification of crucial metabolites

**Figure 6:**
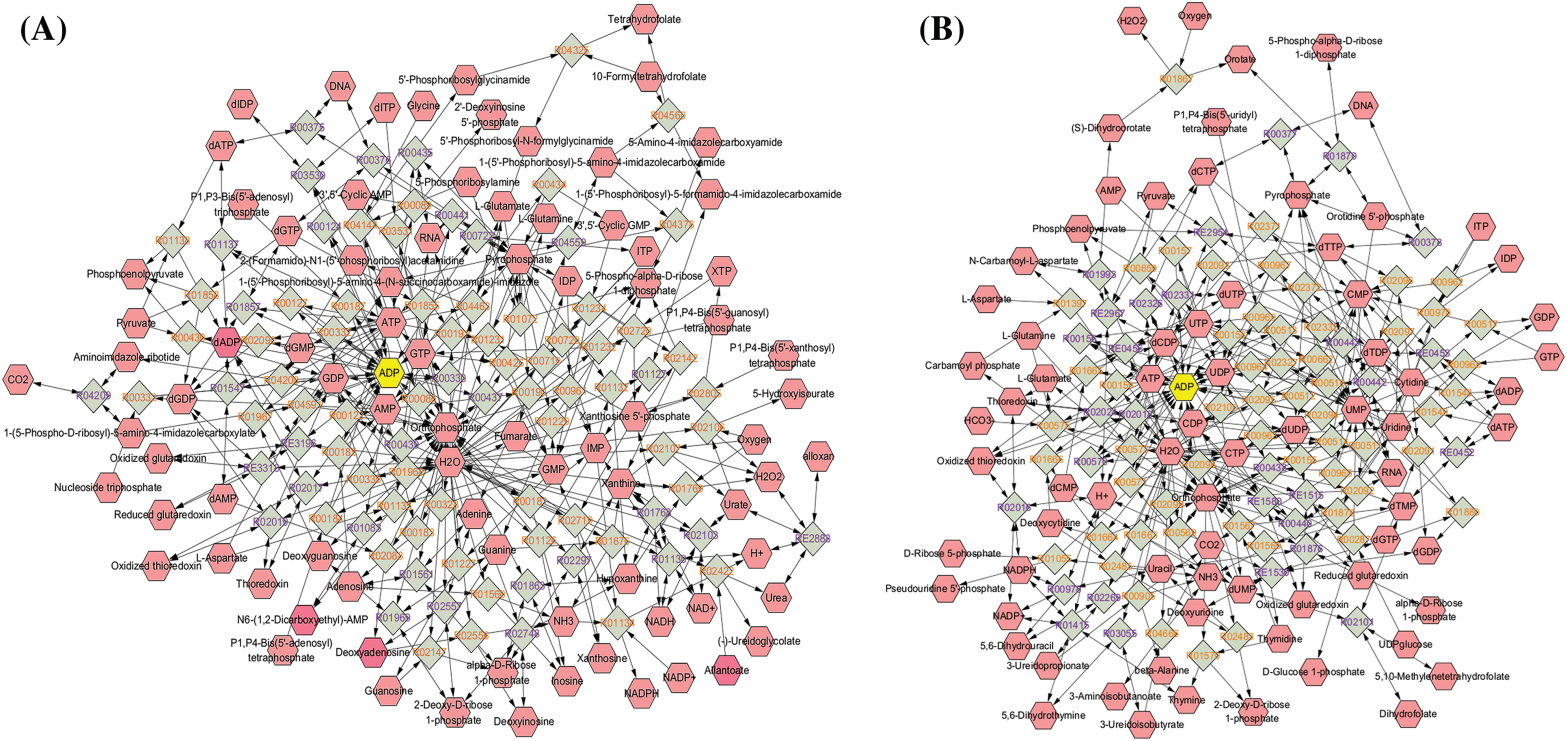
The network diagram showing key compounds. The identified metabolites were imported to Cytoscape, and compound reaction network based on purine metabolism (A) and pyrimidine metabolism (B) were done with the help of Metascape plugin.

To identify key differentially expressed metabolites, we imported the metabolite data to Cytoscape and built a compound reaction network with the help of the Metascape plugin. As shown in Fig. 6A,B, purine nucleotides, including ADP, adenosine monophosphate (AMP), deoxyadenosine diphosphate (dADP), guanosine diphosphate (GDP), and deoxyguanosine monophosphate (dGMP); and pyrimidine nucleotides including CDP, deoxycytidine diphosphate (dCDP), UDP, and deoxyuridine diphosphate (dUDP) were located at the center of the network diagram, especially ADP which was increased in the H460_met group. Intriguingly, a previous study found that nucleotide metabolites in *Lkb1*-null cells, including hypoxanthine nucleotide (IMP), AMP, guanosine monophosphate (GMP), dGMP, uridine monophosphate (UMP), ADP, UDP, CDP, dCDP, and deoxythymidine diphosphate (dTDP), were present at consistently lower levels compared with *Lkb1*-wt cells [27], implying the upregulation of these monophosphate or diphosphate nucleotide metabolites may reinvigorate the immune response against *STK11* mutant NSCLC. Of note, multiple nucleotide metabolites enriched in metabolic pathways, including dTMP, CDP, dADP, Cytidine 5′-diphosphocholine and UDP-N-acetylglucosamine, are upregulated in the H460_met but not H460_KO_met group (Fig. 5F). Coincidently, previous studies found that CDP-ethanolamine pathway is critical for T follicular helper cells differentiation and humoral immunity [28], and lowering of UDP-N-acetylglucosamine levels resulted in c-Myc reduction in NK cells, which require c-Myc for granzyme B expression and cytotoxic activity [29]. These results indicated that these nucleotide metabolites could potentiate anti-tumor immunity.

### The verification of these identified nucleotide metabolites

**Figure 7:**
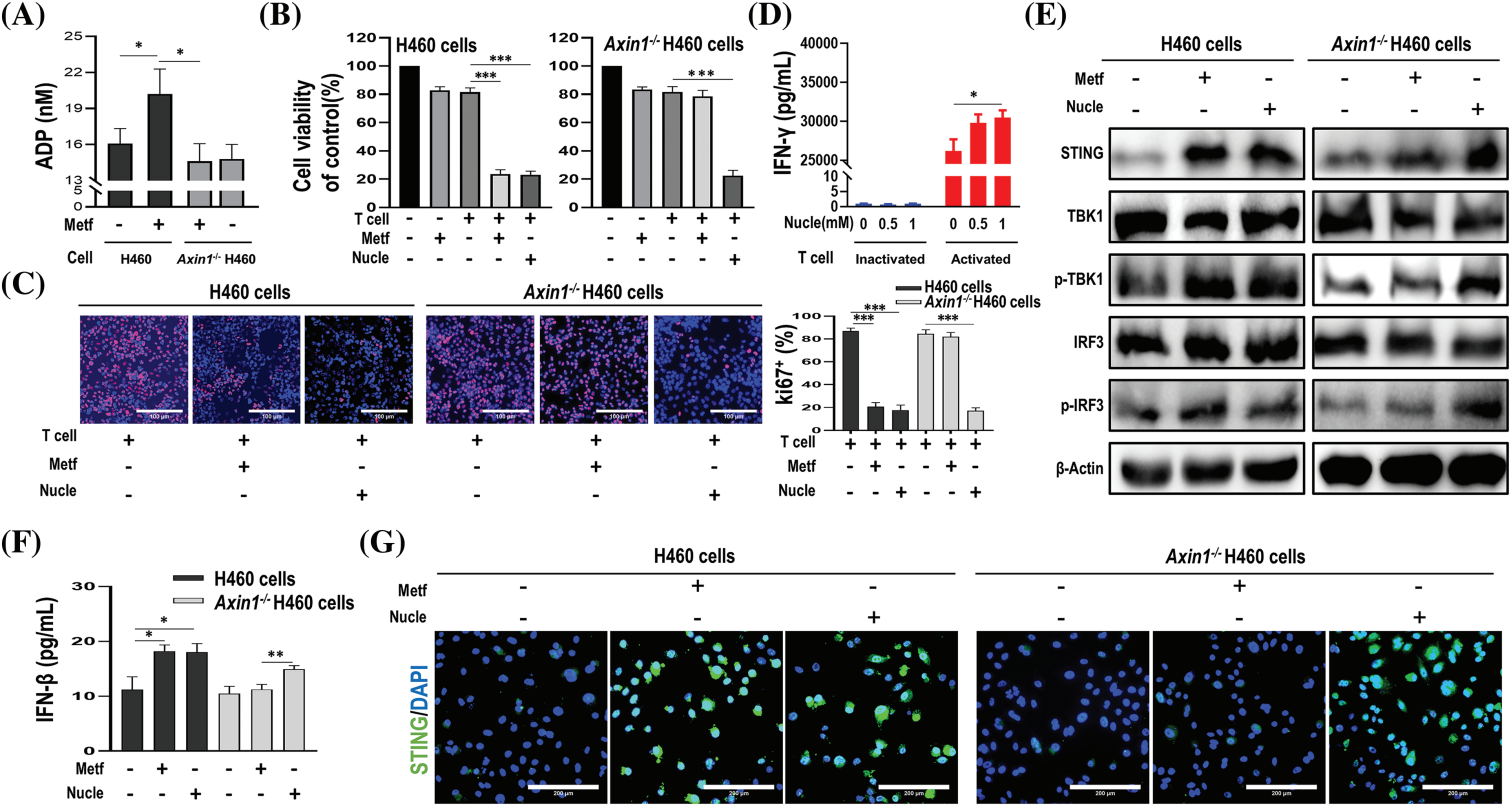
The identified nucleotide compounds could activate STING and enhance anti-tumor immunity. (A) ADP assay for cells treated with or without metformin (1 mM). (B) Cell viability of cells treated with metformin (1 mM), activated T cells (1:1 ratio to cancer cells), or activated T cells combined with metformin or nucleotide compounds (1 mM). (C) Ki67 incorporation assay in H460 cells and *Axin1*^-/-^ H460 cells treated as indicated. Activated T cells (1:1 ratio to cancer cells), metformin or nucleotide compounds were added to the culture medium for 48 h. Cells were then counterstained with DAPI. scale bars: 100 μm. (D) ELISA analysis of IFN-γ in supernatants from inactivated or activated T cell cultures treated with nucleotide compounds (0, 0.5, or 1 mM, respectively) for 48 h. (E) Western blot analysis of indicated proteins in H460 and *Axin1*^-/-^ H460 cells with metformin or nucleotide compounds treatment. (F) ELISA analysis of IFN-β in H460 and *Axin1*^-/-^ H460 cells treated with metformin or nucleotide compounds. (G) Representative immunofluorescent staining of STING in H460 and *Axin1*^-/-^ H460 cells upon metformin or nucleotide compounds administration. Scale bars: 200 μm. Data are presented as mean ± SD of triplicate determinations (for A–D and F). The significant difference was shown as *p < 0.05; **p < 0.01; ***p < 0.001. Metf: metformin; Nucle: nucleotide compounds.

To confirm whether these nucleotide metabolites that were upregulated in metformin-treated H460 cells but down-regulated in metformin-treated *Axin1*^*-/-*^ H460 cells, were involved in metformin-induced anti-tumor immunity, we first examined the production of ADP upon metformin treatment. As shown in Fig. 7A, compared to H460 cells, the level of ADP increased significantly in metformin-treated H460 cells. However, metformin treatment didn’t increase the production of ADP in *Axin1*^*-/-*^ H460 cells. In addition, we performed CCK-8 assay and Ki67 incorporation assay to assess cell viability and proliferation. In agreement with our previous results, metformin or activated T cells alone failed to decrease cell viability, while metformin or nucleotide compounds (a mixture of CDP, ADP, dTMP, and UDP-N-acetylglucosamine) combining with activated T cells significantly decreased H460 cell viability (Fig. 7B left) and the percentage of Ki67-positive cells (Fig. 7C left panel). Furthermore, nucleotide compounds combined with activated T cells could reduce *Axin1*^*-/-*^ H460 cell viability and proliferation, while activated T cells alone or combined with metformin failed to decrease cell viability (Fig. 7B right) and inhibit cell proliferation in *Axin1*^*-/-*^ H460 cells (Fig. 7C right panel). The cytotoxic T-cell killing of tumor cell was partly mediated by the pro-apoptotic effects of IFN-γ, and we previously reported that metformin stimulated IFN-γ production in activated T cells [16,30]. Next, we performed ELISA assay and found that nucleotide compounds treatment in inactivated T cells had little effect on the release of IFN-γ, while it promoted IFN-γ secretion in activated T cells (Fig. 7D). These results indicate that the nucleotide compounds might mediate metformin-induced anti-tumor immunity.

As STING activation is crucial for anti-cancer immune response [18], and we previously found that metformin enhanced the efficacy of immunotherapy in *STK11* mutant lung cancer via AXIN1-based STING stabilization [16], whether the nucleotide compounds involved in the activation of STING were unknown. Thus, we performed western blot to investigate STING expression, as shown in Fig. 7E, the expression of STING and downstream p-TBK1 and p-IRF3 were both increased upon metformin and the nucleotide compounds administration in H460 cells. Meanwhile, nucleotide compounds rather than metformin could activate STING/IRF3 pathway in *Axin1*^-/-^ H460 cells. Next, ELISA assay confirmed that in H460 cells, metformin and the nucleotide compounds have similar effect on the release of IFN-β, a downstream signaling of STING, but only the nucleotide compounds increased IFN-β secretion in *Axin1*^-/-^ H460 cells (Fig. 7F). Furthermore, immunofluorescence staining showed that the level of STING expression was increased in metformin and the nucleotide compounds treated H460 cells, while the nucleotide compounds preserved activation of STING in *Axin1*^-/-^ H460 cells (Fig. 7G). Taken together, through cell experiment, we demonstrated that these nucleotide metabolites enhance anti-tumor immunity by activating STING signaling.

## Discussion

In the present study, we assessed the metabolite profile of H460, H460_met and H460_KO_met via untargeted metabolomics and uncovered distinct metabolites among the three groups. Depending on AXIN1 expression in H460 cells, metformin upregulated multiple nucleotide metabolites through metabolic pathways and promoted anti-tumor immunity in *STK11* mutant NSCLC [31]. Thus, our study indicates that targeting specific nucleotide metabolites and tumor metabolism could provide a potential therapeutic strategy for *STK11* mutant lung cancer.

The current study provides novel insight into how metformin promotes anti-tumor immunity by modulating the TME. These data in combination with a previous study provide precedence for use of metformin to remodel the TME in *STK11* mutant NSCLC [32], which is associated with a dismal prognosis and shows primary resistance to immunotherapy [6]. Notably, it was reported that *STK11* mutations were related to an immunologically “cold” TME, characterized by low PD-L1 expression and decreased tumor-infiltrating CD8^+^ lymphocytes [33,34]. However, very few studies have clarified the metabolic characteristics of the TME in *STK11* mutant NSCLC, with specific alterations in metabolites induced by metformin unclear. Based on our previous study, we further found that distinct metabolic features discriminated against the three treatment groups (Fig. 1A,B) in the present study. Currently, metabolomic analysis of tumor metabolites plays a pivotal role in cancer diagnosis, treatment selection, and efficacy assessment in the era of precision medicine [35]. Therefore, metabolomics has a promising potential to explore differences in the TME to uncover the mechanisms by which metformin promotes immunity against *STK11* mutant NSCLC.

There has been increasing evidence supporting the notion that cancer metabolism is widely implicated in regulating anti-tumor immune responses by releasing metabolites and influencing the expression of immune molecules [36]. To broadly define the metabolomic characteristics among the three groups, we performed untargeted metabolomics which provides the broadest possible metabolome coverage and identified hundreds of metabolites, most of which were annotated into metabolism pathways according to the KEGG and HMDB pathway database (Fig. 2). Although the anti-tumor role of metformin has been widely studied, its impact on tumor metabolism of *STK11* mutant NSCLC and the involved crucial metabolites were unknown. For this reason, we first explored the different characteristics of H460 and H460_met as well as H460_KO_met and H460_met (Fig. 3) groups using metabolomic approaches. Essential metabolite clusters were selected through FCM clustering analysis (Fig. 4). Furthermore, we found that enrichment of metabolic pathways may be crucial for the anti-tumor immunity efficacy induced by metformin (Fig. 5). In consensus with our study, Xu et al. found that the gut microbiome also influenced the efficacy of PD-1 inhibitors in microsatellite stability (MSS)-type colorectal cancer via modulation of metabolic pathways [37]. Additionally, the combination of T-cell therapy and metabolic intervention could enhance solid tumor immunotherapy [38]. These results and the findings from our current study suggest that remodeling the TME by targeting metabolic pathways may be a promising therapeutic strategy for improving immunotherapy. Researches on metabolic reprogramming in cancer using advanced techniques will provide useful insights and novel therapeutic strategies in the future.

As an anti-diabetic agent, metformin’s anti-tumor effects are predominantly through inhibiting oxidative phosphorylation (OXPHOS) via the mitochondria and activating AMPK [21]. By alternating between oxidative phosphorylation and glycolysis, tumor cells can adapt to metabolic challenges [39]. Thus, through targeting tumor metabolism, metformin may be an attractive strategy against cancer. A previous study demonstrated that metformin combined with hypoglycemia impaired tumor metabolic plasticity and growth by modulating the Protein phosphatase 2A (PP2A)-glycogen synthase kinase 3beta (GSK3β)-myeloid cell leukemia 1 (MCL-1) axis [40]. According to KEGG pathway analysis, the metabolic pathways and metabolites enriched in the pathway were identified (Fig. 5E,F), thus broadening our knowledge of metformin’s impact on the metabolism of *STK11* mutant NSCLC, and providing an opportunity for us to explore novel therapeutic strategy by altering metabolites in the TME.

In the current study, we identified 26 possible metabolites that may play a critical role in the process of metformin-mediated anti-tumor immunity. Notably, there were multiple nucleotide metabolites, with most significant variation between the treatment groups (Fig. 5F). Similarly, Liu et al. discovered distinct nucleotide metabolites between *Lkb1*-wt and *Lkb1*-null cells, and demonstrated that metabolites in both purine and pyrimidine metabolism reduced significantly in *Lkb1*-null cells [27]. Furthermore, targeting nucleotide metabolism has been shown to be a promising strategy to enhance cancer immunotherapy [41]. Thus, we performed T cell-mediated killing of cancer cells experiment to verify our findings (Fig. 7B,C). Additionally, the silencing of STING leads to immune escape in *STK11* mutant lung cancer [34], and CDNs such as cyclic dimeric adenosine monophosphate (c-di-AMP) can elicit immune responses [18], whether these nucleotide metabolites have similar effects are unknown. Although we demonstrated that the nucleotide compounds could activate STING/IRF3 pathway independent of AXIN1 (Fig. 7E–G), its underline mechanism needs further exploration. In sum, these results support our findings and demonstrate a unique ability for the nucleotide metabolites to alter the TME.

Although we found that the impact of metformin on tumor metabolism were dependent on AXIN1, the underlying mechanisms of how AXIN1 mediates this process is unclear. In our previous study, AXIN1 served as a platform for binding with STING, and that was enhanced by metformin administration [16]. As a scaffold protein, AXIN1 serves as a platform for proper functioning of multiple proteins [42]. Studies have reported that metformin’s action on lifespan extension was associated with AXIN/LKB1-mediated AMPK phosphorylation and formation of the v-ATPase-AXIN/LKB1-AMPK complex rendered metformin a switch between catabolism and anabolism [22,43]. A previous study which demonstrated that the hierarchical activation of compartmentalized pools of AMPK depends on the severity of nutrient or energy stress, and moderate increases in AMP activated cytosolic AMPK in an AXIN-dependent manner [44]. AMPK regulates mitochondria-dependent catabolic metabolism and plays a vital role in maintaining energy balance [45]. AXIN1 also plays multiple functions in metabolism, including the regulation of catabolic and anabolic pathways and its involvement in AMPK signaling [46]. Shin et al. found that axin expression restrained the activity of OXPHOS complex IV and oxygen consumption rate (OCR), suggesting axin-mediated mitochondrial ATP synthesis in HeLa cells [47]. Furthermore, STING and downstream Type I interferons can cause metabolic reprogramming characterized by reduced glycolytic activity and oxidative phosphorylation (OXPHOS) and increased mitochondrial damage [48]. Therefore, we speculate that the impact of AXIN1 on metabolite alterations may relate to the activation of STING or AMPK.

However, some limitations of this study require further investigation. Firstly, our results predominantly come from metabolomic analysis, which remains uncertain regarding the data’s accuracy. Secondly, we did not explore the distinct metabolite profile between the H460 and H460_KO groups. Additionally, we only conducted cellular experiments to verify these nucleotide metabolites, their role *in vivo* needs to be validated in future studies.

## Conclusion

In summary, the current study demonstrated that metformin promotes anti-tumor immunity in *STK11* mutant NSCLC in an AXIN1-dependent manner through the upregulation of multiple nucleotide metabolites. These findings may provide a therapeutic strategy to overcome immunotherapy resistance in *STK11* mutant NSCLC. Future studies are required to further investigate the role of these metabolites and the mechanism by which AXIN1 affects metformin’s regulation of tumor metabolism.

## Supplementary Materials

Figure S1 **Pearson correlation analysis between quality control (QC) samples.** Pearson correlation between negative (A) and positive (B) QC samples, respectively.

Figure S2 **PLS-DA plot comparison of different groups.** Score plots from the PLS-DA model of H460 and H460_met in negative (A) and positive (B) ion mode, respectively. PLS-DA plots between H460_KO_met and H460_met in negative (C) and positive (D) ion mode, respectively.

Figure S3 **Heatmap illustrating the metabolites patterns and differences between H460 and H460_met group.** Heat maps depict relative abundance of differential metabolites between H460 and H460_met group in negative (A) and positive (B) ion mode.

Figure S4 **Heat maps depict relative abundance of differential metabolites between H460_KO_met and H460_met group in negative ion mode.** The up-regulated (A) and down-regulated (B) metabolites in H460_KO_met group are respectively illustrated.

Figure S5 **Heat maps depict relative abundance of differential metabolites between H460_KO_met and H460_met group in positive ion mode.** The up-regulated (A) and down-regulated (B) metabolites in H460_KO_met group are respectively illustrated.

## Data Availability

The datasets used and analyzed during the current study are available from the corresponding authors on reasonable request.

## Supplementary Materials

The supplementary material is available online at https://doi.org/10.32604/or.2024.052664.
